# The Laboratory Opossum (*Monodelphis domestica*) Is a Unique Model for Research on Zika Virus: Robust Immune Response, Widespread Dissemination, and Long-Term Persistence

**DOI:** 10.3390/v16121847

**Published:** 2024-11-28

**Authors:** André Filipe Pastor, Susan M. Mahaney, Juan Garcia, Marisol Morales, Oscar Quintanilla, Marco A. Arriaga, John M. Thomas, John L. VandeBerg

**Affiliations:** 1 Division of Human Genetics, School of Medicine, The University of Texas Rio Grande Valley, Edinburg/Harlingen/Brownsville, McAllen, TX 78520, USA; susan.mahaney@utrgv.edu (S.M.M.); marco.arriaga01@utrgv.edu (M.A.A.); john.thomas@utrgv.edu (J.M.T.III); 2 Center for Vector-Borne Disease, The University of Texas Rio Grande Valley, Edinburg/Harlingen/Brownsville, McAllen, TX 78520, USA; 3 Sertão Pernambucano Federal Institute of Education, Science, and Technology, Floresta 56400000, Pernambuco, Brazil; 4 South Texas Diabetes and Obesity Institute, School of Medicine, The University of Texas Rio Grande Valley, Edinburg/Harlingen/Brownsville, McAllen, TX 78520, USA; 5 Department of Biology, The University of Texas Rio Grande Valley, Edinburg/Harlingen/Brownsville, McAllen, TX 78520, USAoscar.quintanilla@uhsrgv.com (O.Q.)

**Keywords:** zika virus antibodies, zika virus ELISA, marsupial immunology, flavivirus

## Abstract

The Zika virus (ZIKV) epidemic elicited a rapid commitment to the development of animal models for ZIKV research. Non-human primates (NHPs) and mice have made significant contributions to this research, but NHPs are expensive, have a long gestation period, and are available only in small numbers; non-genetically modified mice are resistant to infection. To address these deficiencies, we have established the laboratory opossum, *Monodelphis domestica*, as a small animal model that complements the mouse and monkey models. We developed and validated an indirect ELISA for measuring antibodies to ZIKV in opossums, as well as an immunohistochemistry (IHC) method to detect ZIKV NS1 protein in tissue samples. Opossum pups inoculated intracerebrally as embryos, juveniles inoculated by several routes, and mothers that cannibalized inoculated pups became persistently infected with ZIKV. The virus spread to multiple organs and persisted for up to 38 weeks (the latest endpoint of the experiments). A robust humoral immune response was mounted, and high titers of antibodies also persisted for 38 weeks. The results establish *M. domestica* as a natural, non-genetically modified animal model in which ZIKV persists long-term after experimental exposure and as a unique animal model for research on the immune response to ZIKV.

## 1. Introduction

Zika virus (ZIKV), an RNA arbovirus belonging to the Flaviviridae family, is transmitted by mosquitoes of the genus *Aedes* and was initially isolated from rhesus macaques in the Zika forest of Uganda in 1947 [1,2]. The most common symptoms of infections in humans are rash, fever, arthralgia, and conjunctivitis. Most patients present only mild and transient disease, but severe neurological complications have been described, including congenital Zika syndrome (CZS) [3] and Guillain–Barré syndrome (GBS) [4]. Newborns with CZS present a distinct clinical phenotype of microcephaly: abnormalities of skull shape and redundancy of the scalp. In addition, these infants may present fetal immobility, distal contractures of the hands and fingers, and foot misplacements [5]. GBS is a rare autoimmune neurological disease, which is increased in frequency among people who have been infected with ZIKV; it causes progressive paralysis of the limbs and muscle weakness [4].

From 1947 to 2016, ZIKV spread to 60 countries and territories in which active ZIKV infection had been reported [6]. In 2016, a significant increase in the number of neonates with microcephaly and other serious disorders was observed in Brazil and believed to be associated with ZIKV infections in utero [7,8], prompting WHO to declare the microcephaly epidemic a “public health emergency of international concern” [9,10]. By November of 2018, 2819 cases related to ZIKV involving microcephaly and other congenital growth and developmental defects were confirmed in Brazil, of which 1843 were concentrated in the Northeast region, including 424 in the state of Pernambuco [11].

The rapid development of this epidemic elicited an immediate and strong commitment to the development of animal models that could be used to develop a better understanding of ZIKV disease, anti-ZIKV immune response, pathologies caused by infection, potential routes of transmission, and efficacy of candidate drugs and vaccines. The primary models that were developed were non-human primates (NHPs) and mice [12,13,14,15,16,17,18]. Both model species have enormous scientific value and potential for research on ZIKV, but they also have severe limitations. The greatest limitations to using NHPs are cost, which prohibits large-scale experimental studies, and the long life cycle and the long gestation period, which do not allow results from developmental and longitudinal studies to be obtained quickly [19]. A major limitation of mice is the fact that wild-type immunocompetent mice are resistant to infection by the virus after the neonatal stage, and neonatal mice experimentally infected with the virus die within days [20]. Furthermore, data obtained from genetically modified immunodeficient mice do not adequately represent normal human subjects for translating experimental results to understanding the biological sequelae of ZIKV infection in humans [21]. Finally, these genetically modified animals do not serve as fully valid models for vaccine development or efficacy testing [21,22]. Although the chicken embryo [23] and guinea pig models [24,25] have been used with limited utility, no animal model that obviates the above-mentioned limitations has been established previously.

In response to the deficiencies of animal models suitable for research on ZIKV, we have established *Monodelphis domestica*, the genetic stocks and strains of which are known as laboratory opossums [26], as a model that complements the NHP and mouse models. This model is especially advantageous for research on exposure to ZIKV in the embryonic and fetal stages of development [27], as well as for research on exposure in fully immunocompetent juveniles and adults. *M. domestica* adults weigh from 80 to 150 g versus 20 to 30 g for mice, so four to five times more blood volume can be safely removed from an opossum compared to a mouse on a single occasion or over a given timeframe. In addition, a substantial amount of blood can be safely removed from the opossums at an earlier stage of development than in mice. By comparison with monkeys, opossums have a short gestation period (14 days, even shorter than mice), produce large litters (a mean of 10 and as many as 13 pups at weaning for the most fecund stock), breed continuously (capable of rearing four litters per year if the litters survive, and many more if the pups are harvested or die pre-weaning), are inexpensive to maintain (in mouse cages; fed commercial pelleted opossum chow), are economical to produce and to use experimentally in large numbers, and reach sexual maturity in 6 months [26].

While these favorable characteristics make laboratory opossums ideal, and in some instances unique, laboratory animals for experimental research in many fields of biology, they also have some additional characteristics that make them unique as animal models for research on ZIKV. Their developmental stage at birth is similar to that of a 5- to 6-week human embryo and to that of a 12-day mouse embryo [28], so they complete most of the embryonic and all of the fetal development outside of the mother’s uterus. Moreover, female *M. domestica* do not have a pouch, so the neonates (each of which attaches to a teat shortly after birth) are easily accessible for experimental manipulation when the mother is anesthetized. In addition, the neonates are extraordinarily robust to experimental manipulation throughout development. They can be inoculated directly with ZIKV beginning on the day of birth, enabling controlled experiments that are not confounded by maternal and placental physiology or by virus interactions with the mother’s immune system. ZIKV can even be inoculated directly into the brain on the day of birth [27]. In addition, *M. domestica* at any life stage can support the long-term persistence of the virus. Moreover, a variety of genetic stocks and strains are available for research on host-virus interactions and on the genetics of immune response to ZKV inoculation and consequent pathologies.

The characteristics of the laboratory opossum make this species uniquely suited as a third important model, together with NHPs and mice, for investigating the biological and pathological sequelae of exposure to ZIKV. The validation of this model makes its use feasible in large-scale experimental protocols of types that are not practical with NHPs or rodents. This capacity may transform fundamental experimental research on host responses to ZIKV and pathologies caused by ZIKV, and it can exert a sustained and powerful influence in this field.

Toward that goal, we developed and validated an indirect ELISA for measuring antibodies to ZIKV in laboratory opossums, as well as an immunohistochemical method to detect ZIKV NS1 protein in tissue samples. We used these methods to determine some basic characteristics of the opossum humoral response to ZIKV lineages from Puerto Rico and Brazil, persistence of antibodies and the virus in animals inoculated at different ages and via different routes, effects of multiple sequential inoculations of individual animals, tissue tropism, the relative efficiencies of single vs. several sequential inoculations and varied routes of inoculation to elicit an antibody response, and anti-ZIKV antibody kinetics up to 38 weeks after exposure to the virus. In addition, the cannibalization of some inoculated pups by their mothers provided an opportunity to explore the possibility of oral transmission of the virus, an event that may be an important factor in establishing and maintaining high levels of viremia in wild *M. domestica* in their natural environment.

## 2. Materials and Methods

### 2.1. Animals

The laboratory opossums were produced in the breeding colony at The University of Texas Rio Grande Valley (UTRGV) and were maintained under standard conditions in individually ventilated cages [26]. Blood samples were collected by cardiac puncture under isoflurane anesthesia. The animals were checked daily for signs of illness. The few animals that became seriously ill were euthanized by CO_2_ inhalation.

### 2.2. Antigens and Preparation of Virus

Four antigens were evaluated for their capacity to capture antibodies against ZIKV. Two of them were the recombinant antigens ZIKV ENV (ZENV16-R-10) and NS1 (ZNS117-R-10) purchased from Alpha Diagnostics Intl. (San Antonio, TX, USA). The other two were ZIKV lineages PRV (Puerto Rican Virus, PRVABC059) and BZV (Brazilian Virus, BR1911), produced in the laboratory of one of the authors (JMT) and inactivated by UV irradiation before use.

Live PRV and BZV were used for inoculations. As per reference [27], for the preparation of the virus, Vero cells (CCL-81; ATCC, Manassas, VA, USA) were used for virus titration, whereas C6/36 cells (CRL-1660; ATCC, USA) were used to amplify the lyophilized virus. Following a single passage in C6/36 cells, the supernatant was clarified and purified using a sucrose cushion. For plaque assays, Vero cells were seeded in six-well plates the night before. A 90% confluent monolayer was infected with tenfold serial dilutions of PRV, incubated for 96 h, fixed with 4% PFA, and stained with crystal violet. Virus supernatants were quantified by plaque assay and stored at −80 °C.

### 2.3. Inoculations and Sample Collection

**Figure 1:**
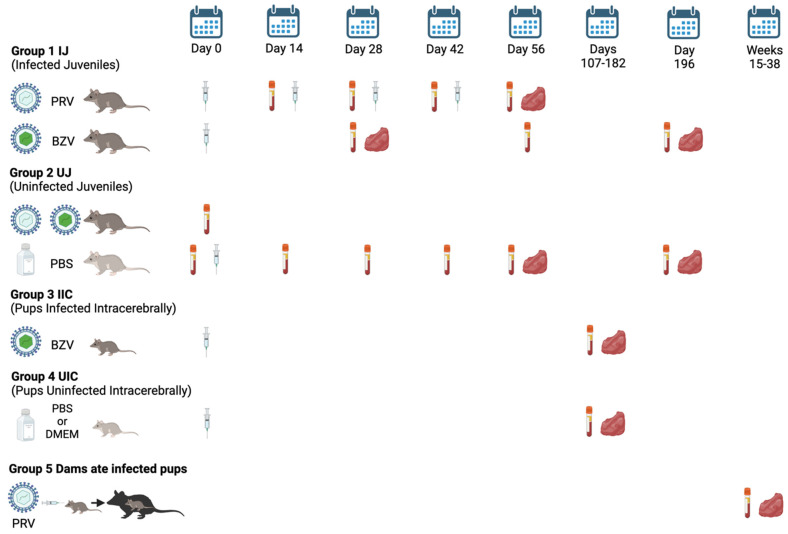
Study design and timeline. There were four groups of animals in the study design: 1. Presumed Infected Juveniles (IJ), which were inoculated with PRV or BZV; 2. Uninfected Juveniles (UJ), from which a baseline blood sample was collected before they became Group IJ animals (**top**) or from which blood was collected after they had been injected with PBS (**bottom**); 3. Pups presumed to be Infected Intracerebrally (IIC) by inoculation of BZV; 4. Pups inoculated intracerebrally with PBS or DMEM, Uninfected Intracerebrally (UIC). In addition, Group 5, Dams (Dams) that ate infected pups, was established when it became apparent that dams that cannibalized inoculated pups in another study might have become infected by oral transmission. The timeline is represented by days after the opossums were injected (syringe) with ZIKV or PBS or DMEM or, for Group 5, by weeks after they ate infected pups. The vacutainer tubes indicate dates of blood collection, and the red symbols indicate dates of tissue harvest.

The study design is shown in Figure 1. Juvenile opossums were inoculated using a 27 g needle with PRV via five routes, including intraheart (IH), intramuscular (IM), intraperitoneal (IP), intratesticular (IT), or subcutaneous (SC), or with BZV via the IM, IP, or SC routes. Each inoculum contained 10^5^ PFU in 50 µL of Dulbecco’s Modified Eagle Medium (DMEM). Control animals were injected with 50 µL of sterile phosphate-buffered saline (PBS) or DMEM.

PRV animals were injected starting at 18 weeks of age, which was designated as Day 0 of the experiment, given three booster shots of ZIKV at 2-week intervals (Days 14, 28, 42), and necropsied for tissue collection at 26 weeks of age (Day 56). Tissue samples were placed in 10% formalin or flash-frozen at −80 °C. Serum samples were collected on Day 0 before the first injection, immediately before each booster, and at the time of necropsy, and stored at −80 °C.

BZV animals were injected at 18 weeks of age only, and serum samples were collected on Days 28, 56, and/or 196 after inoculation. Tissue samples were collected at 22 weeks of age (Day 28) or 46 weeks of age (Day 196).

Suckling pups were injected intracerebrally with 5000 PFU of BZV in 2 μL of DMEM. Control pups were inoculated with sterile PBS (16 animals) or DMEM (16 animals). Inoculations were conducted when the animals were 3–8 days of age, and the animals were euthanized at 22, 26, 29, or 52 weeks of age. One animal, P1967, was euthanized early at 19 weeks because it had an inflamed, bleeding scrotum. Another animal, P1968, was euthanized at 21 weeks of age as an approximately age-matched control for P1967. For data analysis, both animals were placed in the 22-week-age group. At the time of animal processing, blood was collected for isolation of serum, and tissue samples were harvested and placed in 10% formalin or flash-frozen at −80 °C until they were analyzed.

Ten dams unexpectedly cannibalized infected suckling pups that were in a parallel experiment to inoculate litters with PRV instead of BZV, but we have not yet begun to analyze the tissues from the remaining pups in these litters. The dams had been inoculated with 1000 PFU of PRV and were euthanized between 18 and 38 weeks after they ate their pups; blood and tissue samples were collected. An exception was an additional dam (P1110) whose pups were inoculated with a high dose of PRV (10^5^ PFU); that dam was euthanized 15 weeks after she ate nine of her pups. 

### 2.4. Serum Samples

The 503 serum samples available for testing by ELISA to detect antibodies against ZIKV were derived from five groups, each of which represented a different route of potential exposure. Group 1, Infected Juveniles (IJ): two hundred thirty-nine (239) samples were available from 102 potentially infected juveniles that had been inoculated with ZIKV lineage BZV BR1911 (*n* = 59) or ZIKV lineage PRV ABC059 (*n* = 43). Group 2, Uninfected Juveniles (UJ): the negative control group comprised 188 samples collected from the same animals on Day 0 of the study (before inoculation of ZIKV) (*n* = 107) or that were inoculated with placebo (*n* = 81). Group 3, Suckling Pups Infected Intracerebrally (IIC): thirty-three samples were available, one from each of 33 animals that had been inoculated intracerebrally with ZIKV (BZV) at an embryonic stage of development (3 to 8 days after birth). Group 4, Suckling Pups Inoculated with Placebo (designated Uninfected Intracerebrally, UIC): the negative control group comprised 32 samples, one from each of 32 animals inoculated with placebo. Group 5. Mothers that Ate ZIKV-Infected Suckling Pups (designated Dams): eleven samples were available, one from each of 11 mothers that ate one or more sucking pups that had been inoculated with ZIKV.

### 2.5. ELISA Optimization

The process of optimization involved testing many different combinations of assay variables on samples selected with the a priori expectation that they would have antibody levels ranging from none to high. The objective was to identify the optimal combination of conditions, i.e., the conditions that provided the greatest power to discriminate between samples from infected and non-infected animals. After the ELISA was optimized, validation was accomplished by performing the ELISA on the 503 samples that were available in the five categories defined above. These included animals injected with saline and believed to be uninfected, animals inoculated with ZIKV and believed to be infected (some of which were documented by immunohistochemistry to be infected; see below), and animals that might potentially have become infected via a route other than inoculation of ZIKV. The factors that were evaluated during optimization were as follows. 1. Concentrations of capture antigens: four concentrations of the recombinant antigens ZIKV NS1 and E (from 6.25 ng/well to 50 ng/well) and two dilutions of the inactivated virus PRV and BZV (100 and 1000 PFU/well) were evaluated for coating the ELISA plates. 2. Dilutions of opossum serum: five serial dilutions of opossum serum, ranging from 1:12.5 to 1:200, were evaluated. 3. Dilutions of secondary antibodies: three secondary antibody dilutions (1:1000, 1:5000, and 1:10,000) were evaluated. 4. Incubation times: two incubation times (1 h and 2 h) were evaluated both for the binding of the opossum antibodies to the capture antigens and for the binding of the secondary antibodies to the opossum antibodies.

After the preliminary results were obtained, a titration curve was developed with five opossum serum dilutions (1:12.5, 1:25, 1:50, 1:100, and 1:200) using pools of presumed positive and presumed negative samples. The dilution that showed the highest ratio of optical densities for the positive pool versus the negative pool, 1:50, was chosen for use in the optimized protocol.

### 2.6. Indirect ELISA Anti-ZIKV

For the optimized protocol, 96-well Nunc MaxiSorp^®^ flat-bottom plates (Invitrogen by Thermo Scientific, Carlsbad, CA, USA) were coated with 100 PFU of ZIKV PRV lineage per well in carbonate–bicarbonate buffer (0.1 M pH 9.6; SRE0034, Sigma-Aldrich, Inc., St. Louis, MO, USA) and incubated at 4 °C overnight (12–24 h). Then, the plates were washed once with washing buffer (PBS 1×, pH 7.2, supplemented with 0.05% Tween 20—PBS-T). Blocking was performed for 1 h at room temperature using PBS 1×, pH 7.2, plus 1% BSA (BP 1600, Fisher Scientific Co., Pittsburgh, PA, USA), followed by three washes with PBS-T. Then, 100 µL of opossum serum diluted 1:50 in PBS-T supplemented with 1% BSA (PBS-T/B) was loaded into each well, followed by incubation at 37 °C for 2 h. The plates were wrapped with plastic wrap before incubation. After three washes, 100 µL of goat anti-opossum IgG (H + L)-HRP conjugate (Alpha Diagnostic) diluted 1:1000 in PBS-T/B was added to each well, followed by 2 h incubation at 37 °C. Following the final three washes, the TMB substrate (T5525, Sigma-Aldrich, Inc., St. Louis, MO, USA) was added, and the plates were incubated for 15 min at room temperature. Then, the reaction was stopped with 50 µL of 2 M H2SO4 (Sigma 258105). Finally, the plates were read at 450 nm using a Multiskan^TM^ FC Microplate Photometer (Thermo Scientific, Waltham, MA, USA), and the results were expressed as optical density (OD).

### 2.7. Serum Pools and Quality Control

Negative and positive serum pools were included on each ELISA plate. The negative pool was used to calculate the cutoff value that discriminates negative from positive samples, while the purpose of the positive pool was to ensure that the assay had functioned properly in its ability to detect positive samples.

The negative pool was created from equal volumes of serum collected on Day 0 just before ZIKV inoculation from 48 naïve animals at the time the serum was collected; serum from these animals had tested negative during the optimization process. One hundred microliters of this pool, diluted 1:50, was assayed in nine wells of each plate. The positive pool was created from equal volumes of 29 aliquots of serum from animals that had been inoculated as juveniles on one occasion with ZIKV (BZV) 196 days before blood collection, and that tested positive during the optimization process. The positive pool was composed of samples that tested in the low range of positivity to make it a more robust validator of the ELISA capacity to detect low-positive samples. One hundred microliters of this pool, diluted 1:50, were assayed in three wells of each plate.

To test the reproducibility of the ELISA, the coefficient of variability [%CV = (σ/mean OD) × 100] was calculated (σ = standard deviation; OD = optical density). Four groups, spanning the range of ELISA values that had been observed, were used for this calculation: 1, high positive; 2, medium positive; 3, low positive; 4, negative. Groups 1, 2, and 3 were formed by creating a pool from 10 positive samples each, while Group 4 was the same negative pool described above. Five assays were conducted on different days, using four replicates for each group. The intra-assay %CV was calculated based on the mean OD of the five replicates [29].

### 2.8. Immunohistochemistry (IHC)

Tissues analyzed by IHC included brain, eye, heart, spleen, and reproductive organs (testis, epididymis, vagina, and ovary). Seventy-six animals from the five groups (IJ, UC, IIC, UIC, and Dams) were examined. The IHC method follows [27] with some modifications. Tissue sections were incubated in PBSTB (PBS + 0.01% Tween20 + 0.2% BSA) for 1 h, followed by incubation with a 1:500 dilution of primary antibody (Arigo Biolaboratories, Taiwan) for 1 h at room temperature or overnight at 4 °C. The primary antibody targeted the ZIKV NS1 protein. After the primary antibody was removed with three quick washes and three 10 min washes in PBSTB, the slides were incubated in a 1:200 dilution of AlexaFluor 546-conjugated secondary antibody (Invitrogen by Thermo Scientific, Carlsbad, CA, USA) for 1 h. The secondary antibody was removed similarly, with the addition of DAPI and AlexaFluor 488-conjugated phalloidin during the first 10 min wash at 1:1000 and 1:200 dilutions, respectively. Imaging was performed using an Olympus FV10i confocal microscope or a Motic BA410E with a fluorescent upgrade.

Validation of the anti-NS1 antibody signal was conducted by analyzing two tissue slides from each sample: one with the primary antibody and the other without it. The ovary from a ZIKV-infected animal, P2141, served as a positive control, whereas tissues from animals injected with PBS were used as negative controls.

### 2.9. Statistical Analysis

The mean absorbance value for each sample, assayed in triplicate, was used as the measure of serum antibody level against ZIKV. Eight candidate calculations of cutoff values were evaluated for their ability to discriminate between known positive and known negative samples. The one that was best able to discriminate between positive and negative samples was empirically determined to be, for each ELISA plate, the mean OD of the nine wells containing the negative serum pool plus 3× the standard deviation of that mean, multiplied by 1.75. The formula [(Mean OD + 3 × SD of mean OD) × 1.75] defined the cutoff described above. The titer was calculated by dividing the test sample’s mean absorbance by the cutoff value. A serum sample with a titer between 0.900 and 0.999, inclusive, was considered to be indeterminate, while below 0.900 was designated as negative, and equal to or higher than 1.000 was designated as positive [30]. The sensitivity, specificity, and positive and negative predictive values also were determined.

In addition, a receiver operating characteristic (ROC) curve was made to determine the accuracy of the test for juveniles and pups that were inoculated with the virus. In addition, the mean titers of the routes of injection were compared between animals inoculated with BZV or PRV, and the antibody kinetics were evaluated for the days following the inoculations. The variables were compared among the groups using a one-way ANOVA analysis with false discovery rate (FDR) correction with *p* values less than 0.05 were considered statistically significant. Graphs were made using the GraphPad Prism software version 6.0 (GraphPad Software, San Diego, CA, USA).

## 3. Results

### 3.1. ELISA Optimization

**Figure 2:**
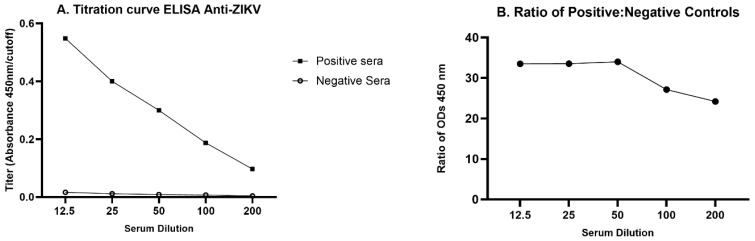
Titration curve for ELISA anti-ZIKV in opossums. Serum pools were diluted serially from 1:12.5 to 1:200. (**A**) The positive serum pool was made from equal volumes from 29 inoculated juveniles, whereas the negative serum pool was made from equal volumes from 48 uninfected juveniles. (**B**) Ratio of OD mean positive/OD mean negative pool at each dilution (means derived from four replicates).

To determine the highest sensitivity of the ELISA for detecting anti-ZIKV in opossums, we first compared serially diluted serum pools from inoculated and presumed infected juvenile opossums and presumed uninfected juvenile controls (Figure 2A). The 1:50 dilution yielded the highest ratio of positive/negative OD (Figure 2B) and was chosen to be used in the optimized protocol. Other optimized parameters were documented to be inactivated PRV as the capture antigen at 100 PFU/well, blocking with 1% BSA and a secondary antibody dilution of 1:1000.

### 3.2. Indirect Anti-ZIKV ELISA

**Figure 3:**
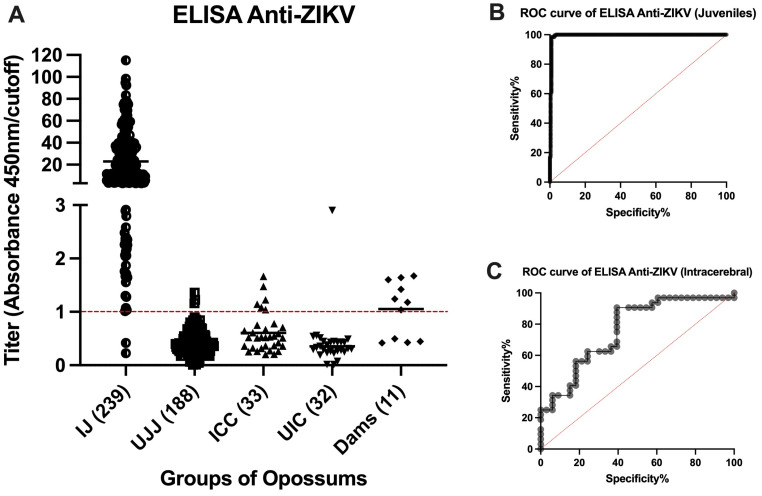
ELISA evaluation of the response of anti-ZIKV antibodies in different groups of infected opossums. (**A**) Summary of the anti-ZIKV ELISA results. The points represent the results of the serum samples from the animals in the various groups evaluated; different symbols represent data points from different groups: IJ: Inoculated Juveniles; UJ: Unexposed Juveniles; IIC: Infected Intracerebrally; UIC: Unexposed Intracerebrally; Dams: Ate inoculated pups. Titers between 0.900 and 0.999, inclusive, are considered indeterminate, whereas those below 0.900 are negative, and those equal to or higher than 1.000 are positive. The horizontal bars represent means. (**B**,**C**) Receiver operating characteristic (ROC) curves evaluating the sensitivity of the ELISA with serum from IJ (**B**) and IIC (**C**) groups. For the IJ group, the area under the curve was 0.9945 (99.45% accuracy), whereas it was 0.7861 (78.61%) for the IIC group.

A total of 237 of the 239 available samples from virus-inoculated juveniles were positive by ELISA, and two were negative (Figure 3A). Both negative samples were from animals that had received a subcutaneous inoculation of ZIKV 2 weeks (O9460) or 4 weeks (P1397) before blood collection, respectively. It had been entered into the record of O9460 that some (or all) of the inoculum was observed to leak out of the injection site immediately after inoculation. The animal O9460 was inoculated subcutaneously again at Days 14, 28, and 42 after the first ZIKV injection and had converted to positive by ELISA in samples collected at Days 28, 42, and 56. No animals inoculated by any other route failed to yield a positive ELISA result at the first or any subsequent blood collection.

A total of 185 of the 188 samples collected from the same animals on Day 0 of the study (before inoculation of ZIKV) or that were inoculated with placebo gave negative ELISA results. However, three samples gave low positive results (Figure 3A). We believe that these three results were false positives. The cutoff value was chosen to minimize false negatives at the risk of having a higher (but still low) number of false positives. These data reflect a sensitivity of 99.14%, specificity of 98.40% (Table 1), and accuracy of 99.45% (Figure 3B).

Six of the 33 samples from animals that had been inoculated intracerebrally with ZIKV (BZV) at an embryonic stage of development (0 to 9 days after birth) were positive by ELISA (Figure 3A); the other 27 samples were negative. Thirty-one of the 32 samples from the negative control group were unequivocally negative by ELISA. The one positive sample was a high positive, suggesting exposure to a high dose of ZIKV. For the intracerebrally inoculated group, if it were assumed that all ZIKV-inoculated animals became infected and developed antibodies to the virus, the sensitivity of the test would be 18.18%, the specificity would be 96.88% (Table 1), and the accuracy would be 78.61% (Figure 3C).

Four of the serum samples from the 11 mothers that ate one or more sucking pups that had been inoculated intracerebrally with ZIKV were negative by ELISA, and seven were positive (63.6%) (Figure 3A).

**Figure 4:**
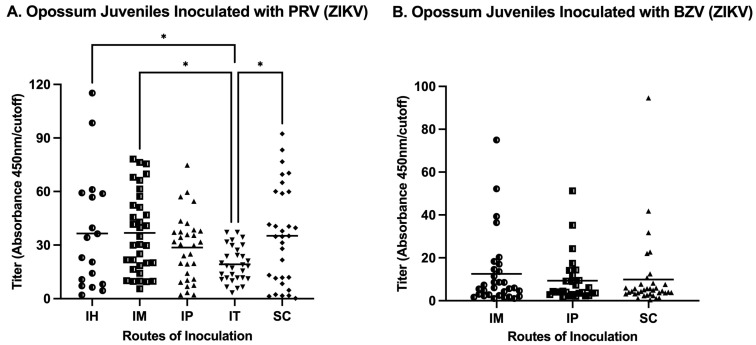
Comparison of titers elicited by different routes of inoculation of juveniles. The horizontal bars represent means. Different symbols represent data points from different groups. (**A**) Routes of inoculation with PRV (IH: Intraheart; IM: Intramuscular; IP: Intraperitoneal; IT: Intratesticular; SC: Subcutaneous). (**B**) Routes of inoculation with BZV (IM: Intramuscular; IP: Intraperitoneal; SC: Subcutaneous). A one-way ANOVA analysis with false discovery rate (FDR) correction was performed. * *p* < 0.05.

The analysis of the routes of injection of juveniles showed that IM inoculations resulted in the maximum mean titer in animals infected either with PRV or BZV (Figure 4). Regarding the mean values for juveniles inoculated with PRV, the IH, IM, and SC routes resulted in statistically significantly higher titers than the mean titer of animals inoculated by the IT route (Figure 4A). For BZV inoculation, although the IM route resulted in a higher mean titer, there was no statistically significant difference among groups inoculated by the IM, IP, or SC routes (Figure 4A).

**Figure 5:**
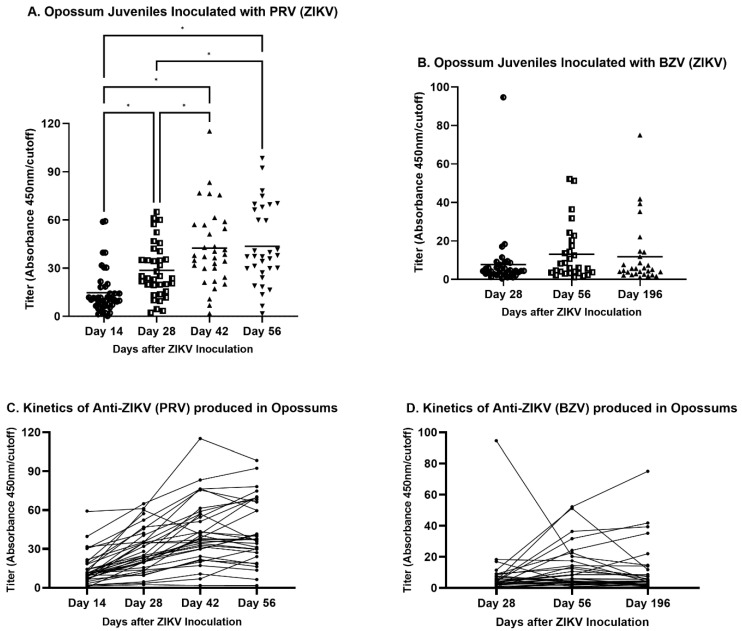
ELISA results for anti-ZIKV antibodies in opossums inoculated with PRV bi-weekly on four occasions or BZV on one occasion. The horizontal bars represent means. Different symbols represent data points from different groups. (**A**,**B**) A one-way ANOVA analysis with false discovery rate (FDR) correction was performed. * *p* < 0.05. (**C**,**D**) Kinetics of viral titers. Each line represents a single animal.

The inoculation schedules differed for PRV and BZV. Animals were inoculated with PRV on four sequential occasions (Days 0, 14, 28, and 42), while opossums were inoculated with BZV only on Day 0 of the study. The titers of animals inoculated with PRV increased sequentially from Day 14 to Day 42, but they did not increase further by Day 56 (Figure 5A). However, BZV-inoculated opossums exhibited no significant differences in titers at 28, 56, or 196 days after inoculation (Figure 5B), although the titers were higher on Days 56 and 196 than on Day 28. The lack of significance may have been a consequence of a single outlying titer at Day 28, with a value that was five-fold the next highest titer at that time point.

The antibody kinetics during the days after inoculation also were assessed for each animal (Figure 5C,D). In PRV-infected animals, the titers increased from Day 14 to Day 42, as the animals received bi-weekly inoculations. After the final inoculation on Day 42, the titers of some animals continued to increase, whereas the titers of other animals decreased (Figure 5C). The kinetic pattern of BZV-infected animals did not change from Day 56 to Day 196, with few exceptions (Figure 5D).

### 3.3. Quality Control

To determine the coefficients of variability of the ELISA, one negative and three positive pools with high, medium, and low titers were tested in four replicates. The intra-assay CV was 16.7%, while the inter-assay variability was 16.1% (Table 2). Coefficients of variation with values less than 20% indicate adequate repeatability of the assay [30].

### 3.4. IHC

#### 3.4.1. IJ and UJ Groups

**Figure 6:**
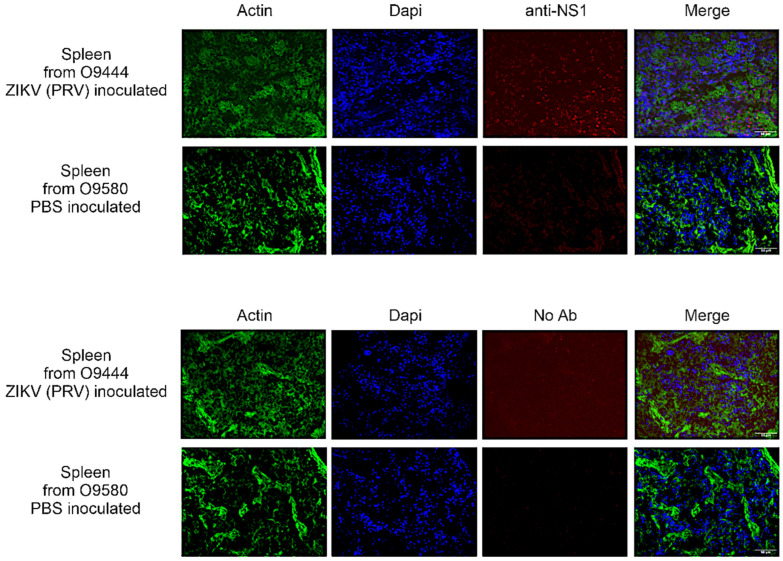
IHC results from spleens of O9444, an animal inoculated with PRV by the IT (intratesticular) route, and O9580, an animal injected with PBS by the IH (intraheart) route and necropsied at 26 weeks of age. Images depict tissue stained with and without primary antibody for detection of viral non-structural protein 1 (NS1). Images were photographed at 40× magnification on three separate channels (green for cytoskeleton, blue for DAPI, and red for NS1 signal) and merged for the composite photo.

To determine the extent of distribution and long-term persistence of ZIKV in infected animals, IHC was conducted on a subset of animals inoculated as juveniles with ZIKV (IJ group) or PBS (UJ) group. Twenty-six animals inoculated with PRV as juveniles were assessed for NS1 by IHC, and the signal was detected in 24 of them in some tissues. NS1 protein was detected in brain, eye, heart, spleen, and/or reproductive organs (Table 3). Representative results from the spleen of an IHC-positive animal (O9444) and an IHC-negative animal (O9580) are shown in Figure 6. Note that slight background staining is visible on the right-hand side of the image of O9580 stained without primary antibody and that diffuse background staining is visible throughout the image of O9444 stained with primary antibody. Those appearances of non-specific background staining are quite distinct from the true NS1 signal in the spleen from O9444, which appears as distinct foci in the anti-NS1 panel and is localized primarily to the nuclei, as is apparent from the merged panel.

The two animals in which no signal was detected in any tissue, O9374 and O9342, had been inoculated by IM and SC routes, respectively. Tissue samples from 12 of the juveniles inoculated with BZV were examined; 10 of them displayed NS1 signals in reproductive organs and/or spleen (Table 3). No signal was detected in two of the animals, P1351 and P1366, which had been inoculated via the IM route. Despite the failure to detect signal by IHC in any tissue of the four animals, all of them were positive by ELISA. P1397 was the only IJ animal that gave a negative ELISA result, but it gave a positive IHC result. Eight juveniles injected with PBS were assessed for NS1 by IHC, and all of them were IHC-negative in all tissues examined. Seven of these control animals were negative in the ELISA; one (O9575) was negative on Study Days 14 and 28 but was positive on Study Days 42 and 56.

#### 3.4.2. Suckling Pups Inoculated Intracerebrally with BZV (IIC Group) or PBS or DMEM (UIC Group)

Seven of sixteen (43.8%) of the IIC animals displayed viral NS1 signal in at least one tissue type, indicating persistence of ZIKV at the time of organ harvesting 26 weeks after inoculation (Table 3). The highest IHC-positive rate was observed in reproductive organs, followed by brain and spleen. No NS1 signal was found in any of the evaluated tissues of the other nine animals. The four UIC animals analyzed did not exhibit any evidence of NS1 protein in the brain, spleen, testes, epididymis, vagina, or ovaries.

#### 3.4.3. Dams

To assess the possibility that ZIKV could be transmitted in this species via the oral route, IHC was conducted on tissues from 10 of the 11 dams that ate suckling pups that had been inoculated with PRV. The dams had eaten their pups 18–38 weeks prior to the collection of tissue samples from the dams. All of the dams displayed an NS1 signal in the spleen. Despite the confirmation that ZIKV was present, anti-ZIKV antibodies were detected in serum by ELISA in only seven of the 11 dams (Table 4). The serum was collected at the same time as the tissues.

### 3.5. Pathologies

In addition to histological abnormalities of brain as well as features of Zika Congenital Syndrome, which were previously reported by us in animals inoculated intracranially as young pups [27], we observed a wide array of pathologies among treated animals in the various groups in the currently reported experiments, including, among others, malformed brain, large or small body size, obesity, absent or extremely small smooth muscle structures of the female reproductive system (uterus and vagina), extremely small ovaries, enlarged (presumably inflamed) uterus and vagina, and abnormal spleen size (large or small), shape, or architecture (involutions on the surface). Only a few treated animals in any group exhibited a pathology, but some exhibited more than one. Examples of multiple pathologies in individual animals included (1) a male that developed an inflamed and bleeding scrotum, necrotic testes, and epididymides, an enlarged spleen, brown (rather than “red”) kidneys, and a pale-colored brain; (2) a female with a small body, an extremely small vagina and uterus, and an unidentified mass in the body cavity; and (3) a female with a malformed brain and an extraordinarily large body. The data on these pathologies and on the weights of the brain and other organs are extensive and complex in relation to the experimental conditions and ages of inoculations of the study groups. When they have been rigorously analyzed, the results and interpretations will be reported in another manuscript.

## 4. Discussion

Our results have established that the laboratory opossum, *M. domestica*, can serve as a unique animal model for research on the humoral response and other sequelae to ZIKV exposure. The combination of characteristics that make it unique are the following: (1) unlike any other model, opossums are born at the developmental stage of a 5- to 6-week human embryo and of an 11.5-day mouse embryo, and ZIKV can disseminate widely and persist long-term after intracerebral inoculation at the embryonic stage; (2) in contrast with the persistence of ZIKV in normal immunocompetent mice and NHPs, ZIKV persists long term in juvenile and adult opossums after inoculation of ZIKV; (3) in contrast with oral transmission in mice and NHPs, ZIKV can easily be transmitted in opossums via the oral route.

For translating the results from opossums to humans, Table 5 relates the ages of the opossums to the approximate ages of developmental equivalency of humans and mice.

### 4.1. Humoral Response to ZIKV

Here, we describe the first indirect ELISA developed to capture IgG antibodies against ZIKV in *M. domestica*. The ELISA results were >99% perfect in detecting true positives among animals inoculated as juveniles with ZIKV; 101 of 102 juveniles inoculated with PRVABC059 or BR1911 mounted a robust humoral immune response against ZIKV (Figure 3). The exception was P1397, but that animal was positive for the NS1 signal by IHC (Table 3). Although P1397, which was inoculated subcutaneously with BZV, might have received a low dose of the virus because of leakage of the inoculated material, the detection of NS1 by IHC confirms that the animal had become infected. Either it failed to mount a humoral immune response, or the ELISA result was a false negative. In the uninfected juvenile group, three of 188 samples showed a low positive signal. We hypothesize that these spurious false positives might have been a consequence of a cross-reaction with a natural antibody of those animals.

P1397 was the only one of the 46 juveniles that was tested by both methods, and that was negative in the ELISA and positive by IHC; five juveniles (P1366, P1351, O9374, O9342, and O9575) that were tested by both methods were positive in the ELISA and negative by IHC (Table 3).

Among the 35 juveniles that were tested by both methods and were positive in the ELISA, P1351, O9374, and P9342 were negative by IHC. Four of those animals were inoculated (and, therefore, were expected to be positive), and one of them (O9575) was injected with PBS and, therefore, expected to be negative. We speculate that the animals that were expected to be positive were most likely infected because, for many animals tested by IHC, some tissues were positive, and others were negative. Apparently, the virus was not present in all tissues of infected animals, or at least it was not present in the sections of the tissues that were examined by IHC. These results suggest that positive IHC results reflect the presence of the virus, but it is clear that negative IHC results in one or more tissue samples do not necessarily reflect the absence of the virus in the animal. The fifth animal that was positive in the ELISA and negative by IHC (O9575) had been injected with PBS; our interpretation is that the ELISA result was a false positive.

Many routes of ZIKV inoculation have been used in ZIKV animal models, such as direct application to palatine tonsils, intraamniotic, intragastric, intranasal, intraperitoneal, intravenous, intrauterine, intravaginal, and subcutaneous [12,14,15,25,31,32]. Subcutaneous is a well-established route used for ZIKV inoculations of guinea pigs, mice, and NHPs [15,19,24]. Rhesus macaques are difficult to infect intranasally, but the application of high-dose ZIKV directly to the tonsils of rhesus macaques resulted in detectable viremia similar to that detected after inoculation by the subcutaneous route [32]. Intranasal inoculation of ZIKV is capable of establishing infection in guinea pigs, A129 immunodeficient mice, and cynomolgus macaques, which were also infected intragastrically [31]. Intravaginal ZIKV infection was successful in the guinea pig model [25]. Almost all of the routes cited above have been utilized to infect immunodeficient mice efficiently [15,31].

Regarding the elicitation of antibodies in mice and other small animal models [33], AG129 mice, which lack interferon receptors a/b and g, produced IgA, IgM, and IgG antibodies against ZIKV. Wild-type C57BL/6 mice also mounted humoral adaptive immune responses to ZIKV, but the responses were not necessary to prevent disease [34]. However, in mice in which the type I IFN pathway was suppressed, the adaptive immune response had an important role in regulating the infection [34]. Virus-like particles (VLPs) containing ZIKV envelope protein domain III also induced potent neutralizing immune responses in C57BL/6 mice [35]. Anti-ZIKV antibodies are documented to have an important response in combating the virus since passive transfer of immune serum significantly reduced viral replication in AG129 mice [36] and was able to prevent the death of wild-type mice inoculated intracerebrally with a lethal dose of ZIKV [16]. Anti-ZIKV neutralizing antibodies were detected in infected Dunkin-Hartley guinea pigs using the plaque reduction neutralization test [24]. Also, ZIKV-specific immunoglobulin G (IgG) antibodies were detected at 14 dpi and sustained thereafter [31]. However, New Zealand White (NZW) rabbits are not susceptible to ZIKV infection, and sera from inoculated animals do not neutralize the virus, indicating a lack of seroconversion [37].

Many species of large mammals, such as goats, lions, sheep, water buffalos, and NHPs, have been demonstrated to produce a humoral immune response against ZIKV [12,22]. Rhesus macaques presented neutralizing antibodies by 21 dpi, and they exhibited no detectable virus replication when challenged 10 weeks after the initial challenge with a homologous strain [19]. In addition, rhesus macaques infected with East African ZIKV were completely protected from detectable viremia when subsequently reinjected with heterologous Asian ZIKV [38]. Moreover, strong anti-ZIKV-specific antibody responses were displayed in both the maternal and fetal/neonatal circulation of rhesus macaques after pregnant females were inoculated [18]. However, adult sheep, cattle, pigs, and chickens are not susceptible to ZIKV infection and do not produce a humoral response against the virus [39].

Our results from juvenile opossums established that all routes (IH: Intraheart; IM: Intramuscular; IP: Intraperitoneal; IT: Intratesticular; SC: Subcutaneous) were efficient in stimulating an antibody response. Anti-ZIKV antibodies were detected 14 days after inoculation (the first timepoint tested) and were present until Day 196 (the last timepoint tested). In summary, the IM and SC routes of inoculation elicited the highest antibody levels (Figure 4A), but the fact that the two samples from animals inoculated by the SC route (which resulted in leakage of the inoculum) were ELISA-negative established that as the IM route is the best route of choice for future investigations involving opossums. The SC route has been predominantly used in NHPs to elicit a humoral response [18,19,38] and may well be appropriate for NHPs and other species, including mice, that have much thicker skin than opossums and that are not prone to leakage of inoculum delivered by that route.

The ELISA identified antibodies in only 18% (6/33) of the pups inoculated intracerebrally, even though ZIKV NS1 protein was detected by IHC in the organs of some of the ELISA-negative animals. A recent study of mice inoculated intracerebrally at embryonic Day 13.5 (E13.5), developmentally similar to an opossum with 2 days of age (Table 5), revealed the up-regulation of immune-related genes 3 days after infection (E16.5) [40]. Similarly, Shao et al. observed that genes related to the immune system were upregulated at E17.5, three days after intracerebral inoculation in mice [41]. These results suggest a strong immune response against ZIKV in mice infected intracerebrally as embryos, at which time infection with ZIKV is associated with abnormal brain development. Our results from opossums suggest that many of the pups inoculated intracerebrally at an embryonic stage were tolerant to ZIKV and were not able to mount an adaptive antibody immune response after their immune systems developed. However, despite the absence of antibodies and the widespread dissemination of the virus to other organs, these animals displayed no signs of illness.

Seven of the 11 dams that cannibalized their pups showed positive ELISA signals (Figure 4). To our knowledge, this was the first documentation in any animal model of ZIKV transmission by eating infected suckling pups, as was confirmed by IHC of the spleens of 10 of the dams. No tissues were available from the eleventh dam, P1110, for analysis by IHC. The apparent absence of antibodies in four of the 11 dams is an enigma. Insight could be gained by conducting ELISA on serial serum samples from opossums exposed orally with different amounts of ZIKV and by conducting IHC on exposed animals at different time points after exposure.

### 4.2. Persistence of ZIKV Infection

Juvenile animals in Group IJ were inoculated at 18 weeks of age with ZIKV isolates PRVABC059 (four bi-weekly inoculations) and BR1911 (one inoculation only); persistence of ZIKV was confirmed by IHC in tissue samples collected at 22–46 weeks of age. The virus was demonstrated to persist for at least 28 weeks (when the animals that had been infected for the longest duration were euthanized), which is equivalent to 9 mouse weeks and 16 human years (Table 5). ZIKV RNA was detected up to 414 days after symptom onset in human semen, indicating that ZIKV also can persist long-term in humans [42].

Intramuscular (IM), intraperitoneal (IP), and subcutaneous (SC) routes of inoculation were conducted with both viral isolates (PRV and BZV); the IHC results established that all three routes were efficient in infecting the animals, that the virus spread to many organs, and that they persisted long-term. Not all animals were assayed by IHC for all organs in the panel used in our study, but among those that were assayed for all organs, a female (O9344) inoculated IP exhibited NS1 in the brain, heart, reproductive organs, and spleen, but not the eye. This result indicated the widespread dissemination and long-term persistence of the virus. All eight control animals injected with PBS (the UJ group) were negative in the IHC assay.

The intracerebral route has been used to determine if ZIKV has the potential to infect the nervous system of mice that are immunocompromised either because they are pre-term or newborn and without a well-developed immune system or are genetically modified [40,41,43,44]. Detection of negative-sense strand viral RNA and isolation of infectious virus confirmed low levels of replicating ZIKV in small foci in the brains of convalescent mice 1 year after they had been inoculated subcutaneously at 1 day of age (before the establishment of a well-developed immune system), but the virus was absent from all other organs [45]. That result from mice is consistent with our IHC results, which established that ZIKV can persist long-term in the brains of infant *M. domestica* (i.e., exteriorized embryos and fetuses) inoculated intracerebrally at 3–8 days of age; however, it is different from our results in that and that the virus spreads from the opossum brain to other organs, where it also persists long-term. IHC detected an NS1 signal only in seven of the 16 animals analyzed (44%) (Table 3). The positive tissues were the brain, reproductive organs of both sexes, and spleen. The fact that organs other than the brain displayed positive signals validates that the animals were infected, that the virus had disseminated, and that it persisted until 26 weeks of age (the endpoint of the study), similar to the developmental stage of mice at 8 weeks of age and humans at 15 years of age (Table 5).

Of the 33 pups infected intracerebrally, 16 animals had evidence of the presence of ZIKV or humoral immune responses. Based on the IHC and ELISA data, we suggest that the 17 samples that were negative in both assays were from animals that never became infected with ZIKV (and did not mount an immune response) (Figure 3 and Table 3). This interpretation is consistent with previous data from our laboratory indicating that not all intracerebrally inoculated opossum pups become infected [27].

Because *M. domestica* is endemic to northeastern Brazil, where the height of the ZIKV epidemic occurred, it is likely, given the persistence of ZIKV after experimental inoculation, that this species is a natural reservoir for ZIKV. If that is the case, *M. domestica* might be much a more important reservoir than NHPs or other eutherian species because the opossums are easily infected with ZIKV at every life stage because the virus disseminates to many organs and persists long-term, and because *M. domestica* are present within and in close proximity to open-air houses in some rural areas. Moreover, we have demonstrated frequent vertical transmission of ZIKV from mothers to offspring in this species (unpublished data), so infected mothers in the wild would be likely to transmit ZIKV to their pups, expanding the reservoir of infected animals. Cannibalism of opossum pups by *M. domestica* mothers occurs frequently, so infected mothers might become super-infected by receiving additional boluses of the virus when they cannibalize infected pups from sequential litters. Although the sylvatic cycle of ZIKV in South America has been discussed regarding wild animals, such as NHPs and mosquitos [46], the concept of opossums serving as reservoirs has not previously been suggested.

## 5. Conclusions

Taken together, our results establish *M. domestica* as a valid animal model that mimics some aspects of ZIKV infection in humans but that also displays some significant differences from humans. The use of opossums is viable in a variety of procedures and protocols that are not possible or practical with other models. We demonstrated that inoculated pups and juveniles could become infected by various routes, that dams that ate inoculated pups became infected, that ZIKV disseminates widely among the body’s organs, and that a robust humoral immune response is elicited. Our results have established this model as an alternative to other models for research on host response to ZIKV and as a unique model for research on some aspects of host response. It also may serve as an alternative model for testing therapeutics and vaccines.

The opossum model provides some research opportunities that overcome the limitations of the mouse and NHP models. Unlike non-genetically modified mouse models, opossums are susceptible to ZIKV infection at every age, and they can be easily and economically inoculated in large numbers beginning at the developmental stage of a 5- to 6-week human embryo. Mice can be genetically modified to make them susceptible to infection after infancy, but genetically modified mice may not be high-fidelity models for translation of results to humans. NHP models are likely to be ideal for direct translation of results to humans, but the long duration of gestation and the limited access to embryos and fetuses in utero are severe impediments. Moreover, it is impossible to use large numbers of NHPs in terminal experiments; a typical group size is four. Therefore, experiments with NHPs have extraordinarily limited ability to discern any but the grossest of differences between groups at a statistically significant level. In addition, the existence of inbred strains and genetically distinct random-bred stocks of opossums offers the opportunity to explore genetic factors that influence the sequelae of ZIKV exposure far beyond what is possible with NHPs. Finally, we point out that in comparative medicine approaches to understanding health and disease, it is often the differences among animal models that lead to an understanding of biological mechanisms; the differences among the opossum, mouse, and NHP models provide opportunities to advance our understanding of mechanisms well beyond the insights that could be gained from only the mouse and NHP models.

Our results lead us to suggest that *M. domestica* might be an important reservoir that contributes to human ZIKV infections in northeastern Brazil, which was the epicenter of the Zika epidemic in 2015–2016, and that, via vertical transmission to pups as well as serial infections of dams that cannibalize pups, opossums might create a long-term risk of new ZIKV epidemics in Brazil. It follows that the Virginia opossum, *Didelphis virginiana*, which is a close relative of *M. domestica* and is densely populated around suburban houses in much of the U.S., might serve as an important reservoir in the U.S.

Future initiatives may include studies to determine viral kinetics, the innate immune response, the production of neutralizing antibodies, and the long-term physical, behavioral, and sexual health of infected *M. domestica*, as well as susceptibility and sequelae of ZIKV infection of *D. virginiana*. In addition, determining how *M. domestica* genes are regulated after the animals are exposed to ZIKV infection could provide insight into the pathways that confer protection from or susceptibility to flavivirus infection. Moreover, additional research on inoculated pups could establish a valuable model of CZS.

The results have demonstrated that laboratory opossums can not only serve as an important third animal model, in addition to mice and NHPs, for research on the sequelae of exposure to ZIKV but that they can be used experimentally to address questions about host-pathogen relationships that cannot be easily addressed, or addressed at all, in mouse or monkey models.

## Figures and Tables

**Table 1:** Summary of ELISA data from the juveniles inoculated by various routes and the pups inoculated intracerebrally. The numbers of positive sera are shown for each group, as well as the sensitivity, specificity, positive predictive value (PPV), negative predictive value (NPV), and accuracy derived from the data based on the comparison between the true positives and true negatives.

Juveniles (IJ and UJ Groups)	*n*	Pups (IIC and UIC Groups)	*n*
**IJ** positives (true positives)	237	**IIC** positives (true positives)	6
**UJ** positives (false positives)	3	**UIC** positives (false positives)	1
**IJ** negatives (false negatives)	2	**IIC** negatives (false negative)	27
**UJ** negatives (true negatives)	184	**UIC** negatives (true negatives)	31
**Statistical analysis**	**%**	**Statistical analysis**	**%**
Sensitivity	99.16	Sensitivity	18.18
Specificity	98.40	Specificity	96.85
PPV	98.75	PPV	85.71
NPV	98.93	NPV	53.45
Accuracy	99.45	Accuracy	78.61

**Table 2:** Results of assessment of intra- and inter-assay variability.

	Intra-Assay CV%	Inter-Assay CV%		
	Mean (%)	SD/Mean OD (%)	Mean OD	SD (σ)
**Positive**				
High	8.5	2.7	0.970	0.026
Medium	11.0	6.8	0.831	0.056
Low	9.9	22.3	0.296	0.066
**Negative**	37.6	32.7	0.007	0.002
**Mean**	**16.7**	**16.1**		

**Table 3:** ELISA and IHC results from all animals for which IHC results are available and that were members of the five groups of opossums: IJ, UJ, IIC, UIC, and DAM. IM: Intramuscular; IP: Intraperitoneal; IC: Intracerebral; SC: Subcutaneous. ELISA results are indicated as positive (+) or negative (−). All organs that were tested are designated by letters in the IHC column: B (brain), E (eye), H (heart), R (reproductive organ), and S (spleen). NA means Not Applicable.

ID Number	Sex	Group	Route	Treatment	Age of Animal at Necropsy (Weeks)	Experimental Study Day	ELISA	IHC	
								Positive	Negative
P1366	F	IJ	IM	BZV	22	28	+		R, S
P1349	F	IJ	IM	BZV	46	196	+	S	R
P1350	F	IJ	IM	BZV	46	196	+	R	
P1351	F	IJ	IM	BZV	46	196	+		R, S
P1424	F	IJ	IM	BZV	46	196	+	R, S	
P1465	F	IJ	IM	BZV	22	28	+	R	
P1341	F	IJ	IP	BZV	46	196	+	S	R
P1475	F	IJ	IP	BZV	22	28	+	R	
P1477	F	IJ	IP	BZV	46	196	+	R	
P1397	F	IJ	SC	BZV	22	28	−	R	
P1426	F	IJ	SC	BZV	46	196	+	R	
P1493	F	IJ	SC	BZV	22	28	+	R	
O9343	F	IJ	IM	PRV	26	56	+	H, R, S	B, E
O9347	F	IJ	IM	PRV	22	28	+	B, R	E, H, S
O9374	M	IJ	IM	PRV	26	56	+		B, E, H, R, S
O9344	F	IJ	IP	PRV	22	28	+	B, H, R, S	E
O9348	F	IJ	IP	PRV	26	56	+	B, R, S	E, H
O9375	M	IJ	IP	PRV	26	56	+	E, R	B, H, S
O9342	F	IJ	SC	PRV	26	56	+		B, E, H, R, S
O9346	F	IJ	SC	PRV	26	56	+	E	B, H, R, S
O9373	M	IJ	SC	PRV	26	56	+	E, R	B, H, S
O9523	F	IJ	IH	PRV	26	56	+	S	
O9530	M	IJ	IH	PRV	26	56	+	S	
O9534	F	IJ	IH	PRV	26	56	+	S	
O9455	F	IJ	IM	PRV	26	56	+	S	
O9461	M	IJ	IM	PRV	26	56	+	S	
O9525	F	IJ	IM	PRV	26	56	+	S	
O9526	F	IJ	IP	PRV	26	56	+	S	
O9443	M	IJ	IP	PRV	26	56	+	S	
O9353	M	IJ	IT	PRV	26	56	+	S	
O9354	M	IJ	IT	PRV	26	56	+	S	
O9376	M	IJ	IT	PRV	26	56	+	S	
O9444	M	IJ	IT	PRV	26	56	+	S	
O9463	M	IJ	IT	PRV	26	56	+	S	
O9524	F	IJ	SC	PRV	26	56	+	S	
O9460	M	IJ	SC	PRV	26	56	+	S	
O9524	F	IJ	SC	PRV	26	56	+	S	
O9539	M	IJ	SC	PRV	26	56	+	S	
O9580	M	UJ	IH	PBS	26	56	−		S
O9572	F	UJ	IM	PBS	26	56	−		B, E, H, R, S
P1542	F	UJ	IM	PBS	46	196	−		R, S
P1330	F	UJ	IP	PBS	22	28	−		R, S
P1521	F	UJ	IP	PBS	46	196	−		R, S
P1540	F	UJ	IP	PBS	46	196	−		R, S
P1541	F	UJ	IP	PBS	46	196	−		S
O9575	F	UJ	SC	PBS	26	56	+		B, E, H, R, S
P1937	M	IIC	IC	BZV	26	177	−	R	
P1965	F	IIC	IC	BZV	26	177	−		B, R, S
P1967	M	IIC	IC	BZV	19	129	−	B, R, S	
P1968	M	IIC	IC	BZV	21	148	−	R	B, S
P2087	F	IIC	IC	BZV	24	163	−		B, R, S
P2090	M	IIC	IC	BZV	24	163	−		B, R, S
P2133	F	IIC	IC	BZV	22	149	−		B, R, S
P2138	M	IIC	IC	BZV	22	149	−		B, R, S
P2141	F	IIC	IC	BZV	22	149	+	B, R	S
P2142	F	IIC	IC	BZV	22	149	+	B, R	S
P2146	M	IIC	IC	BZV	22	149	−	S	
P2241	F	IIC	IC	BZV	22	146	+		B, R, S
P2246	M	IIC	IC	BZV	22	146	−	S	
P2275	F	IIC	IC	BZV	26	177	−		B, R, S
P2276	F	IIC	IC	BZV	26	176	−		B, R, S
P2279	M	IIC	IC	BZV	26	176	−		B, R, S
P1945	F	UIC	IC	PBS	26	175	−		B, R, S
P2296	M	UIC	IC	PBS	26	176	−		B, R, S
P2300	F	UIC	IC	PBS	22	149	−		B, R, S
P2306	M	UIC	IC	PBS	22	149	−		B, R, S
P1456	F	DAM	ate 1 pup	PRV	87	NA	+	S	
P1571	F	DAM	ate 8 pups	PRV	84	NA	+	S	
P1624	F	DAM	ate 2 pups	PRV	83	NA	+	S	
P2217	F	DAM	ate 4 pups	PRV	70	NA	+	S	
P2344	F	DAM	ate 2 pups	PRV	68	NA	−	S	
P2355	F	DAM	ate 10 pups	PRV	67	NA	+	S	
P2375	F	DAM	ate 2 pups	PRV	67	NA	−	S	
P2451	F	DAM	ate 10 pups	PRV	67	NA	−	S	
P2452	F	DAM	ate 6 pups	PRV	67	NA	−	S	
P3132	F	DAM	ate 4 pups	PRV	57	NA	+	S	

**Table 4:** ELISA results from 11 dams whose pups had each been inoculated with either 10^5^ PFU (ID P1110) or 1000 PFU (all other dams) of PRV. IM: Intramuscular; SC: Subcutaneous; IC: Intracerebral. ELISA results are indicated as positive (+) or negative (−), with titers in parentheses. All dams exhibited a strong positive IHC result in spleen samples, except P1110, which was not tested by IHC.

Litter Inoculation Date	Dam ID	ELISA Results	Route	Age of Pups (Days) at Time of Inoculation	Number of Pups Eaten	Number of Pups and Days Between Inoculation and Being Eaten	Number of Weeks Prior to Dam’s Necropsy
9 May 2018	P1110	**+**(1.674)	IC	1	9	1 at 1 day, 1 at 2 days, 1 at 5 days, and 6 at 8 days	14–15
6 November 2018	P1456	**+**(1.036)	IC	5	1	1 pup at 20 days	34
19 October 2018	P1571	**+**(1.241)	IC	1	8	2 pups at 6 days, 3 at 17 days, 3 at 32 days	34–38
1 February 2019	P1624	**+**(1.421)	IC	3	2	1 pup at 18 days, 1 at 33 days	19–21
22 October 2018	P2217	**+**(1.640)	IM	3	4	2 pups at 23 days, 2 at 29 days	34–36
26 November 2018	P2344	**−**(0.425)	SC	4	2	1 pup at 17 days, 1 at 45 days	27–31
3 December 2018	P2355	**+**(1.601)	IM	3	10	5 pups at 14 days, 5 at 18 days	30–31
3 December 2018	P2375	**−**(0.446)	IM	5	2	1 pup at 11 days, 1 at 38 days	27–31
6 November 2018	P2451	**−**(0.419)	IM	4	10	10 pups at 14 days	34
4 February 2019	P2452	**−**(0.496)	SC	4	6	2 pups at 15 days, 3 at 25 days, 1 at 30 days	19–21
11 February 2019	P3132	+(1.180)	IC	4	4	2 pups at 12 days, 1 at 17 days, 1 at 23 days	18–21

**Table 5:** Developmental equivalencies of laboratory opossums, laboratory mice, and humans (adapted from [26]). E = Embryonic age in days (d), weeks (w), months (m), or years (y).

Opossums	Mice	Humans	Life History Event
E13.5 d	E10.5 d	E4-5 w	
**0 d**	**E11.5 d**	**E5-6 w**	**Birth of opossum**
2 d	E13 d	E7 w	
4 d	E15 d	E8-11 w	
6 d	E16.5 d	E12 w	
**14 d**	**0 d**	**E16 w**	**Detachment of opossums from nipples; the birth of a mouse**
21 d	3 d	E20 w	Opossum fur growth well-initiated
**30 d**	**6 d**	**0 d**	**Birth of human**
8 w	3.5 w	3 y	Natural weaning, toddler stage
12 w	4 w	6 y	
18 w	5 w	9 y	
22 w	6 w	12 y	Onset of puberty
26 w	8 w	15 y	Adolescence
52 w	17 w	25 y	Physically and reproductively prime
2 y	1 y	50 y	Loss of female fertility
3 y	23 m	75 y	Elderly
4 y	3 y	100 y	Near maximum lifespan

## Data Availability

The original contributions from the study are included in the article; inquiries may be directed to the corresponding authors.
